# Genetic variability in physiological and agronomic traits of newly developed rice lines under well-watered and water-deficit conditions

**DOI:** 10.1186/s12870-025-07436-3

**Published:** 2025-10-02

**Authors:** Raghda M. Sakran, Mohamed I. Ghazy, Mahmoud M. Gaballah, Fatma A. Hussein, Samah M. Aamer, Hasnaa A. Ghazy, Sobhi F. Lamlom, Ahmed M. Abdelghany

**Affiliations:** 1 Rice Research Department, Field Crops Research Institute, Agricultural Research Center, Giza, 12619 Egypt; 2 Plant Production Department, Faculty of Agriculture Saba Basha, Alexandria University, Alexandria, 21531 Egypt; 3 Crop Science Department, Faculty of Agriculture, Damanhour University, Damanhour, 22516 Egypt

**Keywords:** Rice genotypes, Water stress, Physiological traits, Genetic variability, Multivariate analysis

## Abstract

Water scarcity is a critical environmental stress that profoundly constrains rice growth and productivity. Consequently, the development of drought-tolerant and high-yielding rice genotypes is of paramount importance to sustain rice production and safeguard global food security. This field-based study aimed to evaluate the physiological and agronomic performance of diverse advanced rice breeding lines to identify genotypes with high yield and drought tolerance. A set of 15 newly developed rice lines and three check cultivars were used to assess their physiological and agronomic responses under well-watered (WW) and water-deficit (WD) conditions over two consecutive growing seasons, 2022 and 2023. The results showed highly significant differences (*p* < 0.01) among irrigation regimes, genotypes, and their interactions for all studied traits. Under WD conditions, there was a substantial decrease in chlorophyll content, relative water content (RWC), plant height, grain yield (GY), and yield attributes. On the other hand, leaf rolling, proline content, antioxidant enzyme activities (catalase and peroxidase), and sterility percentage were significantly increased compared to WW conditions. The genotypes L8, Sakha-107, and IRAT-170 had the best performance for chlorophyll content, RWC, and stomatal conductance under WD conditions, respectively. Additionally, L5 and L11 recorded the highest GY under WD conditions. The principal component analysis combined with correlation matrix analysis emphasized that both physiological traits (chlorophyll content, RWC, proline accumulation, peroxidase activity, and catalase activity) and agronomic traits (number of panicles per plant and GY) can be considered as effective selection criteria to improve productivity in rice breeding programs under limited water conditions. Notably, genotypes L5 and L11 demonstrated superior GY performance under water-deficit conditions, representing the most valuable genetic materials for drought-prone environments. These elite genetic resources constitute promising germplasm for introgression into breeding programs to enhance rice's drought resilience and maintain productivity under water-limited conditions.

**Supplementary Information:**

The online version contains supplementary material available at 10.1186/s12870-025-07436-3.

## Introduction

As one of the major crops to feed the human population in general, rice (*Oryza sativa* L.) is an important staple for most of the world population. The fact that the importance of rice production is crucial is attributed to the anticipated global population reaching 10 billion by the year 2058 [1]. Global production of paddy rice, in particular, exceeds 755 million which is brought from the area of 162 million hectares of land every year [2]. Indeed, to feed the population and eliminate hunger, it is necessary to increase the production of high-quality rice by more than 60% [3]. As a global phenomenon, climate change perhaps displays an increasingly threatening argument with the agriculture sector since the frequency and intensity of climatic fluctuation issues seem to have escalated pending threats from developing countries. This, in turn, will result in a set of challenges for plants both biotic and abiotic [4]. Such abiotic variables have significant effects on the growth and yield of rice. Rice is facing extreme danger with the drought stress and there is great progress on yield losses [5–7]. Less water availability is a major reason for a lesser yield of rice crops, which is further explained by its semi-aquatic nature and lowland paddy cultivation with a continuous supply of water in the fields during all its stages, making rice one of the most sensitive crops to water scarcity as compared to other cereals [8].

Drought stress affects plant systems through complex physiological mechanisms, with the severity of impact determined by specific environmental conditions. As the primary abiotic constraint in rice cultivation, drought stress significantly reduces both grain yield (GY) and grain quality parameters [9, 10]. Because rice plants are sensitive to water deficit, the tests of newly developed rice genotypes under water deficit and well-watered (WW) environments facilitate the selection of more productive and higher quality cultivars even under unfavorable water conditions [11]. Under drought conditions, rice yields experience significant reductions, with studies documenting yield losses ranging from 15 to 70% depending on the growth stage, stress duration, and severity of water deficit [12]. The reproductive stage has been identified as particularly sensitive to drought stress, where water deficiency can lead to yield losses exceeding 50% due to reduced spikelet fertility, increased spikelet sterility, and compromised grain filling. For instance, research indicates that short spans of drought stress during the reproductive phase can drastically diminish panicle length and seed setting, ultimately resulting in substantial yield reductions [13]. The physiological impacts of drought during critical growth stages have been well documented. Drought stress at the flowering stage has been shown to impair anther dehiscence and pollen viability, leading to increased spikelet sterility and reduced grain number [14]. Additionally, the economic implications of these yield losses are profound. It is estimated that the economic impact of drought stress on rice production could reach billions of dollars annually, particularly in regions where rice is a staple crop [15].

Furthermore, under drought conditions, plants are exposed to osmotic stress, which disturbs their water relations causing a decrease in the efficient utilization of water [16, 17]. Plants have evolved numerous adaptive mechanisms to withstand drought stress, the accumulation of compatible solutes, including proline, is one of the strategies to mitigate the impact of drought stress [18]. Proline accumulation plays a major role in the growth regulation in plants at the time of drought stress [19]. Previous studies have revealed that an increase in proline accumulation in plants results in more drought tolerance and oxidative stress resistance by regulating the antioxidant enzyme activities [20]. It is understood that the antioxidant defense system of plant cells includes enzymatic and non-enzymatic parts. As studies have shown, the activity of antioxidant enzymes might be promoted by drought stress such as catalase and ascorbate peroxidase [21, 22]. The essential presence of these antioxidant enzymes is indispensable for scavenging reactive oxygen species in plant cells [23]. Taken together, plants exhibit a different response to drought stress by amplifying the components of their antioxidant defense system [24].

This study aimed to unravel the complex relationships among physiological traits and yield components under varied water regimes. Besides, a comprehensive comparative approach was used to investigate various physiological (e.g., chlorophyll content, antioxidant enzyme activities, and proline accumulation) and agronomic attributes at different water stress conditions in this study. The novelty of the current investigation resides in the elaborate assessment conducted with a range of newly derived lines for advanced rice breeding. Employing a comparative approach to different water conditions also extends previous studies on drought tolerance. This observation could aid in developing drought-tolerant rice varieties suitable for cultivation in water-limited environments. The objectives of this study were to: 1) investigate the physiological and agronomic responses of 15 advanced rice breeding lines and 3 check cultivars under WW and WD conditions; 2) identify rice genotypes with a consistently high-yielding ability and drought tolerance; 3) determine the relationship among the evaluated traits under WW and WD conditions.

## Materials and methods

### Experimental site and plant materials

**Fig. 1:**
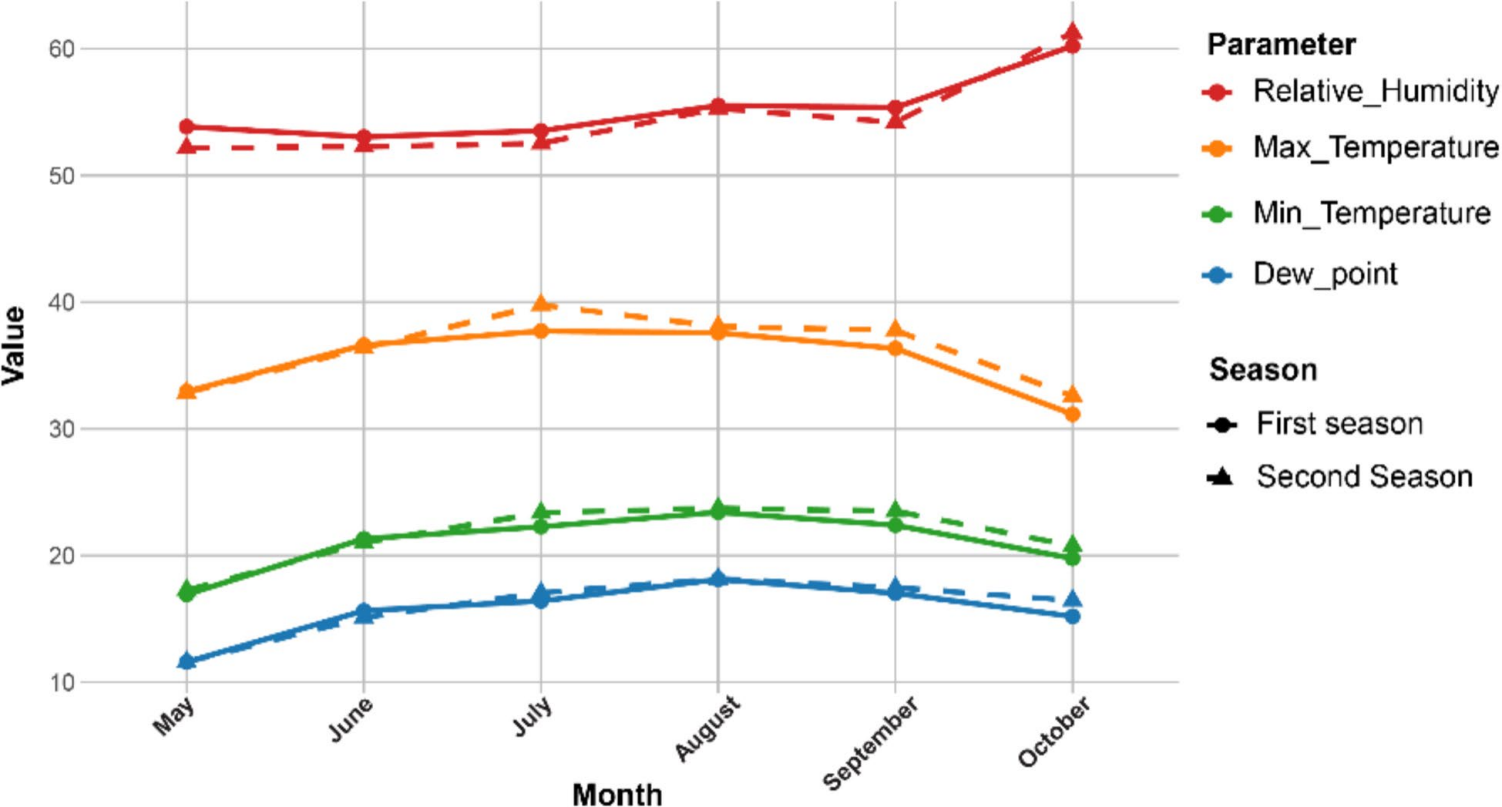
Monthly trends in climatic parameters for rice cultivation across two growing seasons. The graph depicts variations in dew point (°C), maximum temperature (°C), minimum temperature (°C), and relative humidity (%) from May to October in the two growing seasons. Solid lines with circular points represent the first season, while dashed lines with triangular points indicate the second season

The field experiment was conducted at the farm of Sakha Research Station, Agricultural Research Center, Egypt (31° 09' N, 30° 09' E) during the summer seasons of 2022 and 2023. The growing summer season in Egypt is characterized by hot weather with no precipitation events. The meteorological data of the experimental site are illustrated in Fig. 1. Moreover, the soil characteristics of the experimental site during both seasons are shown in Table S1. The soil analysis revealed that the soil was clay throughout the profile (21.33% sand, 29.86% silt, and 48.83% clay). Fifteen newly developed advanced rice genotypes alongside three check genotypes (Table 1) were evaluated under WD and WW conditions in separate experiments during the 2022 and 2023 rice growing seasons. The advanced lines were collected from F_8_ generation in the breeding program of Sakha Research Station following a pedigree scheme.

**Table 1:** Code, name, and parentage of the evaluated rice genotypes

Code	Genotypes	Pedigree
Advanced Lines		
L1	GZ 8452–4-1–1-1	GZ 5603–3-2–2-1/Yun Len 4
L2	GZ 8452–6-1–3-2	GZ 5603–3-2–2-1/Yun Len 4
L3	GZ 8714–7-1–1-2	Giza 177/Aniung Byeo
L4	GZ 9724–11-2–1-2	Reiho/HZ 94- 5
L5	GZ 9730–1-1–1-1	Giza 159/Milyang 23
L6	GZ 9730–1-1–1-2	Giza 159/Milyang 23
L7	GZ 9730–1-1–3-2	Giza 159/Milyang 23
L8	GZ 9781–3-2–2-6	GZ6910-28–1-3–1/Nanjing 15
L9	GZ 9792–13-1–1-2	GZ 6522–15-1–1-13/BL −1
L10	GZ 10239–1-4–4-3	Nabatat Asmar x Skc23822-3–304-3–1-1–1
L11	GZ 9399–4-1–1-3–2-2	Giza 178 × IR 65844
L12	GZ 10739–1-3–2-1	Giza177/KEHWA 4
L13	GZ 10739–1-3–2-3	Giza177/KEHWA 4
L14	GZ 10764–2-2–1-2	Sakha 102 × WAB881SG9
L15	GZ 10778–10-2–2-1	Sakha 104 × IRAT170
Checks		
C1	Giza −177	Giza 171/Yomji No.1//Pi No.4
C2	Sakha −107	Giza 177/BLI
C5	IRAT-170	IRAT13/Palawan

### Experimental design and agronomic practices

Seeds of each genotype were sown in the nursery on the 5th of May in both seasons and transplanted to the field 30 days later. The seedlings of each genotype were individually transplanted in six 5-m long rows with 20 cm space between rows and 20 cm space between hills. A randomized complete block design (RCBD) with three replications was used in each experiment (Fig. S1). The irrigation amounts for each treatment were quantified using a calibrated flow meter. The well-watered (full irrigation) treatment involved maintaining constant flooding conditions, with irrigation applied every four days to ensure a sufficient depth of submersion. This treatment delivered a total irrigation volume of 13,000 m^3^/ha over the course of the growing season. In contrast, the water-deficit (drought) treatment was implemented by irrigating every twelve days, avoiding standing water, and providing a total irrigation volume of 8,500 m^3^/ha. The water-deficit conditions were initiated 15 days after transplantation and sustained until the plants reached maturity. Nitrogen fertilizer was added in three splits while phosphorus and potassium were added in full doses at the time of sowing. Nitrogen fertilizer at a rate of 166 kg N/ha was added in the form of urea (46.0% N). Phosphorous was applied at a rate of 75 kg P_2_O_5_/ha as super-phosphate (15% P_2_O_5_), and potassium at a rate of 90 kg K_2_O kg/ha as potassium sulfate (48% K_2_O).

### Seasonal variations in climatic parameters across two rice growing seasons

The climatic parameters for rice growing across the first season in 2022 and the second season in 2023 reveal distinct trends (Fig. 1). The dew point for the first season ranged from approximately 11.6°C in May to around 15.2°C in November, while the second season started similarly at 11.6°C in May and increased slightly to about 16.5°C by November. Both seasons showed an overall increase in dew point from May through August, peaking slightly higher in the second season. The maximum temperatures for the first season varied from about 32.95°C in May to a peak of around 37.72°C in July, then decreased to approximately 31.16°C in October. In contrast, the second season experienced higher maximum temperatures, starting at 32.86°C in May, peaking at around 39.78°C in July, and dropping to 32.60°C in October. The second season also exhibited slightly higher maximum temperatures than the first season. Relative humidity in the first season started at around 53.85% in May, slightly fluctuating through the months, and increased to about 60.22% in October. The second season began at 52.17% in May, with similar fluctuations, ending at 61.27% in October. Relative humidity remained relatively consistent between the two seasons, with a noticeable slight increase towards the end of the year. These climatic parameters show that the second season experienced slightly higher temperatures and similar humidity levels compared to the first season.

### Measured traits

#### Physiological measurements

Physiological parameters were measured at the heading stage, coinciding with the emergence of the panicle from the flag leaf sheath [25]. This developmental stage was chosen as the measurement point due to its critical and drought-sensitive nature in rice development. During this period, water deficit can substantially affect yield components, and physiological responses serve as effective indicators of the plant's stress adaptation mechanisms. For all physiological parameters, measurements were collected from three biological replicates per genotype per treatment, corresponding to the three replicated plots in our RCBD experimental design. Within each replicate, five plants per plot were sampled, and their average value was used for statistical analysis.

##### Chlorophyll a, b, and carotenoid

Chlorophyll *a, b,* and carotenoid (mg g^−1^ FW) were recorded at the heading stage following the method of [26]. Five fresh leaves were first washed to remove impurities. Then, 2 g of leaf tissue was homogenized in 80% acetone using a mortar and pestle. The homogenates were centrifuged, and the supernatants were used to measure absorbance at 663, 645, and 470 nm with a spectrophotometer. Using these readings, the concentrations (mg g^−1^ FW) of chlorophyll a, chlorophyll b, and carotenoids were calculated.

##### **Proline Content (PC, mg g**^−1^** FW)**

Proline content was assayed according to the method described by [27]. In brief, a plant sample (0.5 g) was extracted in 5% (w/v) sulfosalicylic acid, followed by centrifugation at 10,000 × g for 7 min. The supernatants were diluted with water, mixed with 2% ninhydrin, heated at 94 °C for 30 min, and then cooled. Toluene was then added to the mixture, and the upper aqueous phase was spectrophotometrically assayed at 520 nm.

##### Antioxidant enzymes activity

A fresh leaf weighing 0.5 g was homogenized in ice-cold 0.1 M phosphate buffer (pH 7.5) with 1 mM EDTA-Na2 and 7.5% polyvinyl pyrrolidone. After centrifugation at 15,000 × g for 15 min, the supernatant was collected as the enzyme extract. Catalase (CAT) activity was measured at 240 nm using a spectrophotometer, following the method by Aebi [28]. Peroxidase (POD) activity was determined by monitoring absorbance changes at 470 nm every 3 min, following the procedure of Hammerschmidt et al. [29].

##### Relative Water Content (RWC %)

Relative water content (RWC) was determined as outlined by [30] using the following formula: RWC = [(FW – DW)/(TW – DW)] × 100, where FW is fresh weight, DW is dry weight, and TW is turgid weight.

##### Leaf rolling (LR)

Leaf rolling was determined as an indicator of the degree of drought tolerance on a scale of 1–9 from plant leaves. It was measured by visual estimation following the method of [31].

##### **Stomatal conductance (SC,mmol m**^**−2**^**s**^**−1**^**)**

The stomatal conductance was measured on fully expanded upper canopy leaves between 10 and 12 h with a portable photosynthesis measurement system (Li-Cor, Lincoln, NE, USA) according to [32].

#### Agronomic traits

The number of days to heading (DTH) was recorded as the number of days from transplanting to the date when approximately 50% of panicles in a plot were visually assessed to be fully exerted. Plant height (PH, cm) was measured on 10 randomly selected plants per plot as the distance from the soil surface to the tip of the main panicle at maturity. Panicle length (PL, cm) was measured on 10 randomly selected plants per plot as the distance from the panicle base to the tip of the main panicle at maturity. The number of panicles per plant (NPP) was recorded by counting the total panicles of 10 randomly selected plants per plot. The 1000-grain weight (TGW, g) was determined by weighing 1000 grains randomly sampled from the harvested grains of each plot. Sterility percentage (%) was calculated as the ratio of unfilled grains to total grains from 10 randomly selected panicles per plot. Grain yield (GY, t/ha) was determined by harvesting the four central rows of each plot and converting the yield based on the harvested area.

### Statistical analysis

The collected data for all measured characteristics were analyzed using analysis of variance (ANOVA) with SAS 9.4 software (SAS Institute Inc., Cary, NC, USA). The normality distribution of the residuals and homogeneity of variances were assessed before analyzing variance using the Shapiro–Wilk and Bartlett's tests [33, 34]. The least significant difference (LSD) test (*p* < 0.05 and < 0.01) was used to compare the variations between the irrigation regime, genotype, and their interaction. All figures were generated using RStudio 4.1.1 (Integrated Development for RStudio, Inc., Boston, USA, www.rstudio.com). The weather data across the two growing seasons were visualized with the *ggplot2* package [35]. Principal Component Analysis (PCA) was conducted using the *FactoExtra* package [36]. For Pearson correlation analysis, the packages *ggplot2*, *reshape2* [37], and *RColorBrewer* [38] were employed to conduct and visualize the correlation diagram. For path analysis, the *lavaan* package [39] was utilized to specify and fit the structural equation model, while the *semPlot* package [40] was used to visualize the path diagram.

## Results

### Analysis of variance for physiological and agronomic traits

Data of 16 physiological and agronomic traits measured on 18 rice genotypes which were examined under contrasting water scenarios, WW and drought stress conditions, was subjected to analysis of variance for two consecutive cropping seasons of 2022 and 2023 (Table 2). The findings revealed significant differences among each factor of the genotypes, environments, and their interactions for most traits. For the physiological characteristics, significant differences were found among environments (E) and genotypes (G) (*p* < 0.01) for chlorophyll a and b, carotenoids, catalase and peroxidase, proline, RWC, LR and SC. Significant G × E interactions were found for most traits: chlorophyll a and b, carotenoids, catalase and peroxidase, RWC, LR, and SC. Only proline showed significant differences for E and G (*p* < 0.01) but no G × E interaction. For the agronomic and yield traits, significant differences were found among E and G (*p* < 0.01) for all traits: DTH, PH, NPP, PL, sterility percentage, 1000-grain weight, and GY. Significant G × E interactions were found for most traits: PH (*p* < 0.01), NPP (*p* < 0.05), PL (*p* < 0.05), 1000-grain weight (*p* < 0.01), and GY (*p* < 0.01). DTH and sterility percentage showed significant differences for E and G (*p* < 0.01) but no G × E interaction. GY showed significant differences among years (Y) (*p* < 0.05) in addition to E and G.

**Table 2:** Analysis of variance for evaluated traits of the assessed rice genotypes under well-watered and drought stress over two seasons of 2022 and 2023

Source of Variance	df	Chlorophyll a (mg g^−1^ FW)	Chlorophyll b (mg g^−1^ FW)	Carotenoids (mg g^−1^ FW)	Catalase (Unit mg/protein)	Peroxidase (Unit mg/protein)	Proline content (mg g^−1^ FW)	Relative water content	Leaf rolling
Years (Y)	1	0.03 ns	3.89^**^	0.003 ns	58.47 ns	379.11 ns	0.02 ns	151.74 ns	40.79 ns
Rep/Y	4	0.273	0.03	0.004	13.756	118.194	0.20	57.621	4.339
Environments (E)	1	40.40^**^	18.84^**^	0.27^**^	3426.94^**^	20004.99^**^	2.08^*^	9320.99^**^	39.79^*^
Y x E	1	0.03 ns	0.27^*^	0.02^**^	747.79^**^	2445.68^*^	0.00 ns	14.90 ns	50.90^**^
Error a	4	0.14	0.02	0.003	3.30	162.97	0.19	17.65	2.05
Genotypes	17	0.89^**^	0.76^**^	0.06^**^	82.65^**^	854.29^**^	0.05^**^	93.12^**^	5.89^**^
G x Y	17	0.03 ns	0.34^**^	0.00 ns	5.74^**^	11.10 ns	0.001 ns	7.87 ns	0.75^**^
G x E	17	0.43^**^	0.42^**^	0.02^**^	32.75^**^	419.32^**^	0.03^**^	40.65^**^	3.26^**^
G x Y x E	17	0.07^**^	0.07^**^	0.001 ns	3.25 ns	73.46 ns	0.001 ns	7.77 ns	1.72^**^
Error b	136	0.02	0.01	0.002	2.73	51.87	0.001	16.45	0.27
Source of Variance	df	Stomatal conductance (mmol m^−2^ s^−1^)	Days to 50% heading	Plant Height (cm)	No. of panicles per plant	Panicle length (cm)	Sterility (%)	1000-grain weight (g)	Grain yield (t ha^−1^)
Years (Y)	1	0.001 ns	34.53 ns	295.71 ns	42.05 ns	2.15 ns	18.23 ns	108.80 ns	3.27*
Rep/Y	4	0.002	22.156	270.072	17.542	7.62	12.456	93.837	0.253
Environments (E)	1	0.01^**^	776.87^**^	26,231.8^**^	601.87^**^	258.81^**^	2531.83^**^	757.88^**^	320.99^**^
Y x E	1	0.001^**^	5.47 ns	197.48 ns	129.36^*^	1.42 ns	7.64 ns	45.38 ns	1.62 ns
Error a	4	0.0002	20.41	69.56	6.76	6.76	12.03	32.12	0.23
Genotypes	17	0.002^**^	250.00^**^	1585.24^**^	51.38^**^	58.84^**^	73.13^**^	22.96^**^	15.30^**^
G x Y	17	0.0001 ns	6.13 ns	78.03 ns	7.68^*^	1.16 ns	4.79^*^	4.92 ns	0.15 ns
G x E	17	0.001^**^	7.73 ns	155.85^**^	10.42^**^	3.59^*^	44.95^**^	17.25^**^	5.57^**^
G x Y x E	17	0.00 ns	3.88 ns	71.62 ns	7.35^*^	6.05^**^	3.91 ns	12.30 ns	0.13 ns
Error b	136	0.0001	5.73	48.14	4.03	1.88	2.57	7.37	0.11

ns, ^*^, ^**^ indicate nonsignificant, *p* < 0.05, and *p* < 0.01

### Mean performance of agronomic and physiological traits of rice genotypes under well-watered and water-deficit conditions

The average performance of the 18 rice genotypes was assessed in both WW and WD conditions across the two growing seasons (2022 and 2023) for the physiological characteristics (Table 3). Regarding chlorophyll content, chlorophyll a ranged from 2.39 to 3.36 mg g^−1^ FW for WW conditions, and 1.50 to 2.92 mg g^−1^ FW under drought conditions. Line L5 had the highest chlorophyll a when watered normally at 3.36 mg g^−1^ FW, and L8 had the highest when subjected to drought conditions at 2.92 mg g^−1^ FW. Chlorophyll b was similar, with values from 1.60 to 2.67 mg g^−1^ FW for WW conditions and 0.94 to 2.18 mg g^−1^ FW for WD conditions. Again, L5 and L8 did best for normal and drought stress conditions, respectively. For carotenoids, the average was 0.65 to 0.93 mg g^−1^ FW under WW conditions and 0.60 to 0.88 mg g^−1^ FW under WD conditions. Line L1 had the most carotenoids under WW at 0.93 mg g^−1^ FW, and Sakha-107 did best in under WD at 0.88 mg g^−1^ FW.

**Table 3:** Mean performance of the evaluated 18 genotypes for the physiological traits under well-watered and water-deficit conditions over two seasons of 2022 and 2023

Genotype	Chlorophyll a		Chlorophyll b		Carotenoids		Catalase		Peroxidase		Proline content		Relative water content (%)		Leaf rolling	
	(mg g^−1^ FW)		(mg g^−1^ FW)		(mg g^−1^ FW)		(Unit mg/protein)		(Unit mg/protein)		(mg g^−1^ FW)					
	WW	WD	WW	WD	WW	WD	WW	WD	WW	WD	WW	WD	WW	WD	WW	WD
L1	2.76cd	1.70f	2.14b	1.44cd	0.93ab	0.75bc	26.4cd	32.24de	134.04bc	151.97bc	0.48d	0.73bc	83.93ab	71.25cd	2.03ef	5.04a
L2	2.93bc	1.78ef	2.04bc	1.00f	0.88bc	0.64d	25.72d	33.43cd	113.69d	145.04c	0.44d	0.72bc	83.67ab	69.24d	2.83cd	3.02cd
L3	3.08ab	1.84ef	2.11b	0.94f	0.79c	0.73bc	30.29ab	35.73bc	129.55c	146.58c	0.47d	0.75b	84.62a	67.24de	3.04c	4.04b
L4	2.70cd	1.76ef	1.60e	1.24de	0.65d	0.66cd	27.4cd	32.83de	120.1cd	128.67d	0.45d	0.62cd	77.97cd	69.49d	3.53b	4.02b
L5	3.36a	2.31c	2.67a	1.58c	0.89bc	0.75bc	21.75e	37.64b	126.81c	164.84ab	0.47d	0.87a	83.08ab	71.07cd	3.07c	3.38c
L6	3.23ab	2.30c	2.22b	1.38cd	0.82c	0.76bc	21.36e	30.54ef	131.62bc	150.44bc	0.57c	0.79ab	83.6ab	71.12cd	3.07c	3.23cd
L7	2.61d	2.40bc	1.88cd	1.41cd	0.67d	0.85ab	21.58e	30.43ef	122.88cd	140.39cd	0.58c	0.67c	83.91ab	73.26c	4.78a	4.89a
L8	3.14ab	2.92a	2.33b	2.18a	0.90ab	0.83ab	29.97ab	35.19bc	142.58ab	156.27abc	0.74a	0.80ab	79.43c	67.41de	2.10ef	2.30e
L9	2.91bc	1.50f	2.22b	1.13ef	0.84bc	0.66cd	29.42b	33.94cd	125.65cd	130.91d	0.37e	0.75b	83.27ab	66.50e	3.06c	4.08b
L10	2.85bc	2.25cd	2.00bc	1.59c	0.86bc	0.78bc	29.17bc	33.94cd	136.29bc	144.67c	0.57c	0.73bc	84.67a	70.00cd	4.01ab	4.12b
L11	2.72cd	2.69ab	1.71de	1.70b	0.91ab	0.84ab	20.76e	34.68c	127.82c	137.80cd	0.61bc	0.73bc	87.83a	76.57b	3.02c	3.08cd
L12	3.15ab	2.26c	1.83cd	1.63bc	0.89bc	0.76bc	29.48b	36.03bc	125.64cd	136.44cd	0.60bc	0.78ab	84.95a	73.00c	3.02c	3.26cd
L13	3.08ab	1.88ef	2.37b	1.36cd	0.68d	0.60d	26.95cd	31.98de	106.61d	126.42d	0.47d	0.79ab	83.55ab	68.80de	3.06c	3.12cd
L14	2.39d	1.65f	1.72de	1.31de	0.77c	0.71c	22.38e	34.14cd	126.06cd	146.67c	0.48d	0.61cd	80.82bc	64.94e	3.05c	3.69bc
L15	3.32a	2.48bc	2.30b	2.07a	0.90ab	0.86a	20.57e	27.93f	133.41bc	150.57bc	0.67ab	0.82ab	81.77bc	69.21d	4.15ab	4.48ab
Giza-177	2.94bc	1.81ef	2.26b	1.55c	0.85bc	0.83ab	26.57cd	32.61de	129.13c	136.76cd	0.46d	0.61cd	85.31a	64.26e	2.37de	3.76bc
Sakha-107	3.19ab	2.16cd	2.34b	2.06a	0.92ab	0.88a	27.14cd	36.24bc	107.61d	155.83abc	0.62bc	0.73bc	88.67a	76.79b	1.36f	2.88d
IRAT-170	2.81cd	1.91de	2.13b	1.67bc	0.87bc	0.85ab	28.00bc	38.77a	128.25c	163.95ab	0.61bc	0.71bc	75.95d	72.33c	1.36f	3.74bc
LSD 0.05	0.17		0.16		0.05		1.89		8.22		0.04		4.63		0.6	

LSD_0.05_: Least significant difference indicating whether means in each column are statistically significant at 5% level of significance. Means followed by the same letter within each column are not significantly different

*WW* Well-watered conditions, *WD* Water-deficit conditions

Regarding antioxidant enzyme activities, the levels of catalase activity ranged from 20.57 to 30.29 Unit mg^−1^ protein under WW conditions and from 27.93 to 38.77 Unit mg^−1^ protein under WD conditions. Genotype L3 showed the highest activity under WW conditions, while IRAT-170 was most active under WD conditions. Peroxidase activity ranged from 106.61 to 142.58 Unit mg^−1^ protein under WW conditions and from 126.42 to 164.84 Unit mg^−1^ protein under WD conditions. L8 and L5 showed the greatest levels of activity in wet and dry conditions, respectively. Proline concentration varied between 0.37 and 0.74 mg g^−1^ FW in WW conditions and between 0.61 and 0.87 mg g^−1^ FW in WD conditions. Genotype L8 had the highest proline levels under WW conditions, while L5 had the highest proline levels in the WD environment.

The average RWC ranged from 75.95% to 88.67% in WW conditions and from 64.26% to 76.79% in WD conditions. Sakha-107 showed the highest RWC in both contrasting water conditions, demonstrating its exceptional ability to retain water. LR scores varied between 1.36 and 4.78 with WW conditions, and between 2.30 and 5.76 with WD conditions. Sakha-107 and IRAT-170 had the lowest LR scores in WW conditions, whereas L8 had the least LR in WD conditions.

Furthermore, the average performance of 18 different rice genotypes was assessed for agronomic and yield-attributed characteristics in both WW and water-deficient conditions across two consecutive growing seasons (2022 and 2023) (Table 4). To facilitate comparison of genotype performance under stress conditions, we compiled a comprehensive ranking of all 18 genotypes for agronomic traits under water-deficit conditions (Table S2).

**Table 4:** Mean performance of the evaluated 18 genotypes for the agronomic traits under well-watered (WW) and water-deficit (WD) conditions over two seasons of 2022 and 2023

Genotype	Stomatal conductance (mmol m^−2^ s^−1^)		Days to 50% heading		Plant height (cm)		No. of panicles per plant		Panicle length (cm)		Sterility (%)		1000-grain weight		Grain yield (t ha^−1^)	
	WW	WD	WW	WD	WW	WD	WW	WD	WW	WD	WW	WD	WW	WD	WW	WD
L1	0.07a	0.05b	94.82d	92.81c	84.13e	68.79f	19.82b	16.31d	20.87b	17.10d	4.35c	10.29b	28.88a	24.05b	10.51b	7.68d
L2	0.07a	0.06a	99.69b	93.67c	104.83b	69.78f	24.25a	19.32b	25.49a	21.73b	3.22d	12.17b	27.93a	24.20b	12.76a	7.90d
L3	0.07a	0.06a	95.62d	93.62c	106.66b	78.78d	24.59a	22.48a	18.54c	16.80d	3.02d	5.76e	25.65b	23.13b	11.88a	8.59c
L4	0.05b	0.04c	103.69b	99.68b	85.14e	71.80e	20.87b	17.96c	21.32b	18.84c	5.51b	9.70c	24.88b	23.45b	8.74c	8.05d
L5	0.05b	0.03d	102.98b	97.90b	101.43b	84.62c	19.84b	18.61c	22.06b	20.62b	3.17d	9.66c	26.82a	22.73b	12.15a	9.63b
L6	0.04c	0.03d	98.92c	95.87c	99.25c	75.85d	19.05c	16.06d	20.84b	18.64c	3.35d	9.94c	28.27a	23.03b	11.67a	8.88c
L7	0.05b	0.03d	95.92d	96.94c	101.11b	73.33e	20.50b	18.81c	18.10c	15.11e	2.78e	8.98c	26.73a	21.02c	9.09c	7.34d
L8	0.05b	0.04c	107.07a	101.98a	92.07d	74.03e	17.15d	15.63d	23.84a	21.50b	5.78b	4.04e	26.72a	25.27b	8.86c	8.19d
L9	0.04c	0.03d	97.98c	94.93c	103.13b	87.96b	19.79b	17.51c	19.47c	18.34c	3.24d	9.92c	24.80b	23.05b	9.03c	8.03d
L10	0.04c	0.03d	104.50b	98.50b	104.74b	81.73c	21.40b	18.48c	21.60b	19.87c	4.22c	11.25b	22.80b	19.8c	8.88c	7.89d
L11	0.05b	0.03d	99.33b	99.33b	84.75e	76.35d	19.39b	15.30d	24.75a	23.24a	3.15d	4.64e	26.40a	24.72b	10.60b	9.88b
L12	0.05b	0.04c	97.60c	93.60c	102.95b	76.61d	20.34b	14.94d	21.50b	20.09b	3.20d	9.50c	26.60a	21.90c	10.73b	7.20d
L13	0.05b	0.04c	100.83b	94.75c	93.29d	75.22d	19.59b	13.95e	21.08b	19.72c	3.34d	12.82b	26.17a	21.82c	9.78c	6.13e
L14	0.04c	0.03d	108.07a	106.34a	100.21c	72.25e	18.45c	14.15e	22.70b	17.50d	3.10d	15.36a	27.15a	21.92c	9.36c	4.65f
L15	0.04c	0.03d	99.72b	97.70b	125.36a	106.38a	18.00c	16.98d	23.76a	21.75b	3.56d	12.76b	26.20a	23.15b	11.36a	8.14d
Giza-177	0.07a	0.06a	93.19d	87.11d	108.10b	74.70e	20.27b	12.33e	18.53c	17.21d	5.94b	21.18a	29.10a	17.37d	9.77c	6.63e
Sakha-107	0.06a	0.06a	92.18d	88.13d	113.43a	89.06b	23.04a	18.69c	21.57b	20.11b	4.96c	14.88a	28.28a	25.37b	11.72a	9.21b
IRAT-170	0.06a	0.08a	105.74a	99.99b	133.89a	106.50a	16.88d	15.64d	25.18a	23.62a	8.96a	15.23a	24.90b	22.78b	8.29c	7.28d
LSD 0.05	0.003		5.97		7.92		2.29		1.56		1.83		3.1		0.38	

LSD_0.05_: Least significant difference indicating whether means in each column are statistically significant at 5% level of significance. Means followed by the same letter within each column are not significantly different

*WW* Well-watered conditions, *WD* Water-deficit conditions

Grain yield, the most critical parameter for growers, ranged from 8.29 to 12.76 t ha⁻^1^ under WW conditions and from 4.65 to 9.88 t ha⁻^1^ under water deficit conditions. L2 genotype achieved the highest yield under WW conditions (12.76 t ha⁻^1^), whereas L11 demonstrated superior performance under WD conditions (9.88 t ha⁻^1^), followed by L5, establishing these genotypes as the most valuable for drought-prone environments.

SC varied between 0.04 and 0.07 mmol m⁻^2^ s⁻^1^ in WW conditions and between 0.03 and 0.08 mmol m⁻^2^ s⁻^1^ in WD conditions. Genotypes L1, L2, and Giza-177 showed the greatest SC in WW conditions, whereas IRAT-170 had the highest level in WD conditions. The time taken for 50% heading ranged from 92.18 to 108.07 days with water well conditions and 87.11 to 106.34 days under WD conditions. Genotype L14 took the longest to reach 50% heading in both WW and WD conditions, whereas Sakha-107 was the quickest under both conditions.

PH ranged from 84.13 to 133.89 cm with WW conditions and from 68.79 to 106.50 cm with water deficit conditions. IRAT-170 displayed the tallest plants in both WW and WD conditions, whereas line L1 had the shortest plants in both scenarios. Regarding PL, measurements varied from 18.10 to 25.49 cm in WW conditions and from 15.11 to 23.62 cm in WD conditions. The L2 genotype exhibited the longest panicles in WW conditions, whereas IRAT-170 maintained the longest panicles in WD conditions. The NPP ranged from 16.88 to 24.59 in WW conditions and from 12.33 to 22.48 in WD conditions. The L3 genotype showed the highest NPP in both WW and WD conditions, whereas IRAT-170 and Giza-177 had the lowest NPP in WW and WD conditions, respectively. Regarding sterility percentages, the average performance ranged from 2.78% to 8.96% in WW conditions and from 4.04% to 21.18% in WD conditions. IRAT-170 displayed the greatest sterility during WW conditions, whereas Giza-177 exhibited the highest sterility during WD conditions. Genotype L7 showed the least sterility in WW conditions, while L8 had the lowest sterility in WD conditions.

Regarding 1000-grain weight, values varied from 22.80 to 29.10 g in WW conditions and from 17.37 to 25.37 g in WD conditions. While Giza-177 showed the greatest 1000-grain weight in WW conditions (29.10 g), this genotype performed poorly in actual grain yield, highlighting that grain weight alone may not reflect overall productivity. Sakha-107 exhibited the highest 1000-grain weight in WD conditions (25.37 g) and demonstrated better yield performance compared to Giza-177. A comparative ranking of thousand-grain weight and grain yield for all 18 genotypes under water-deficit conditions is presented in Fig. S2.

### Genetic variability for physiological, agronomic, and yield traits under well-watered and drought conditions

Genetic parameters for physiological traits were assessed under WW and WD conditions (Table 5). Genotypic (δ^2^G) and phenotypic (δ^2^P)variances, along with genotypic and phenotypic coefficients of variation (GCV and PCV), were determined for each trait. Traits such as chlorophyll a and b, peroxidase, and RWC exhibited increased genotypic and phenotypic variances from WW to WD conditions. In contrast, catalase and LR showed a decrease, while carotenoids and proline levels remained consistent across both water scenarios. Except for PC and LR, all physiological traits demonstrated increased GCV and PCV estimates under WD conditions.

**Table 5:** Genetic parameters for physiological, agronomic, and yield-related traits in rice genotypes under well-watered and water deficit conditions evaluated over two growing seasons (2022 and 2023)

Parameter	Chlorophyll a (mg g^−1^ FW)		Chlorophyll b (mg g^−1^ FW)		Carotenoids (mg g^−1^ FW)		Catalase (Unit mg/protein)		Peroxidase (Unit mg/protein)		Proline content (mg g^−1^ FW)		Relative water content (%)		Leaf rolling		
	*WW	WD	WW	WD	WW	WD	WW	WD	WW	WD	WW	WD	WW	WD	WW		WD
Genotypic variance (δ^2^ G)	0.06	0.15	0.04	0.09	0.01	0.01	11.43	6.31	80.80	117.37	0.01	0.01	8.44	11.25	0.64	0.47	
Phenotypic variance (δ^2^ P)	0.07	0.16	0.08	0.12	0.02	0.02	12.08	7.16	86.67	125.60	0.01	0.01	10.18	12.11	0.78	0.74	
Genotypic coefficient of variance	8.05	18.36	9.87	19.29	10.53	10.80	13.09	7.43	7.13	7.46	17.60	9.69	3.49	4.78	27.26	18.13	
Phenotypic coefficient of variance	8.84	18.70	13.12	22.93	10.63	11.03	13.46	7.92	7.39	7.72	17.78	9.81	3.83	4.96	30.10	22.73	
Parameter	Stomatal conductance (mmol m^−2^ s^−1^)		Days to 50% heading		Plant height (cm)		No. of paniclesper plant		Panicle length (cm)		Sterility (%)		1000-grain weight (g)		Grain yield (t ha^−1^)		
	WW	WD	WW	WD	WW	WD	WW	WD	WW	WD	WW	WD	WW	WD	WW	WD	
Genotypic variance (δ^2^ G)	0.0001	0.0002	21.27	20.02	159.66	105.23	2.74	5.05	4.35	4.85	1.96	16.29	1.32	2.52	1.81	1.52	
Phenotypic variance (δ^2^ P)	0.0002	0.0003	22.06	20.90	167.54	122.32	4.51	5.79	5.04	5.36	2.47	17.23	2.59	4.11	1.91	1.57	
Genotypic coefficient of variance	20.77	34.37	4.62	4.65	12.33	12.75	8.20	13.35	9.60	11.27	33.66	36.67	4.32	6.95	13.07	15.71	
Phenotypic coefficient of variance	20.83	34.43	4.70	4.76	12.63	13.75	10.53	14.29	10.33	11.85	37.84	37.71	6.06	8.88	13.42	15.98	

^*^WW Well-watered conditions, *WD* Water deficit conditions

For chlorophyll a and chlorophyll b, genotypic variance increased from 0.06 and 0.04 (WW) to 0.15 and 09 (WD), respectively, with GCV rising from 8.05 and 9.87% to 18.36 and 19.29%, respectively. Carotenoid content showed minimal change in genotypic variance (0.01 for both conditions), while GCV slightly increased from 10.53% (WW) to 10.80% (WD). Catalase activity exhibited a decrease in genotypic variance from 11.43 (WW) to 6.31 (WD), whereas peroxidase activity increased significantly from 80.80 (WW) to 117.37 (WD). PC exhibited stable genotypic variance (0.01) but a decrease in GCV from 17.60% (WW) to 9.69% (WD). RWC showed increased genetic variability under drought, with genotypic variance rising from 8.44 (WW) to 11.25 (WD) and GCV increasing from 3.49% to 4.78%. LR displayed a slight decrease in genotypic variance under drought (0.64 to 0.47), with GCV decreasing from 27.26% (WW) to 18.13%. For SC, a slight increase was noticed in in genotypic variance from 0.0001 (WW) to 0.0002 (WD), with phenotypic variance following the same pattern. The GCV increased substantially from 20.77% to 34.37% under drought, with PCV showing a similar trend (20.83% to 34.43%).

For agronomic and yield traits (Table 5), DTH showed a minor decrease, from 21.27 to 20.02, in genotypic variance under WD conditions, with stable GCV and PCV. PH exhibited reduced genetic variability, with δ^2^G decreasing from 159.66 to 105.23 and phenotypic variance from 167.54 to 122.32, though GCV and PCV slightly increased. The NPP showed increased genetic variability under WD conditions, with δ^2^G rising from 2.74 to 5.05 and GCV from 8.20% to 13.35%. PL displayed slight increases in δ^2^G (4.35 to 4.85) and GCV (9.60% to 11.27%). Sterility percentage demonstrated a substantial increase in genetic variability under drought, with δ2G rising from 1.96 to 16.29 and GCV from 33.66% to 36.67%. 1000-grain weight also showed increased genetic variability, with δ^2^G rising from 1.32 to 2.52 and GCV from 4.32% to 6.95%. GY exhibited a slight decrease in δ^2^G under drought (1.81 to 1.52), but both GCV and PCV increased under stress (GCV: 13.07% to 15.71%).

### Multivariate analysis of genotype performance and trait relationships under contrasting water regimes

**Fig. 2:**
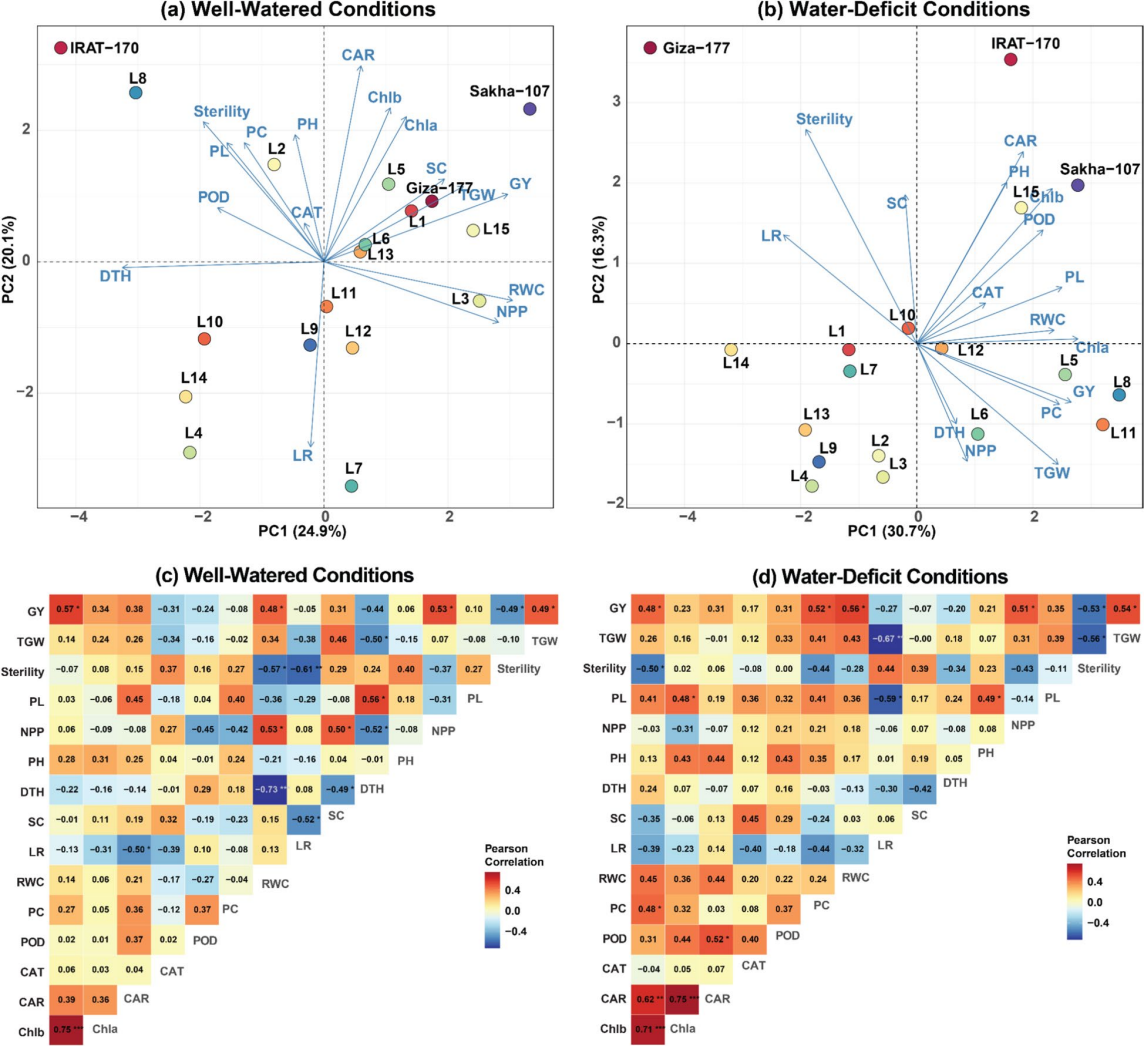
Multivariate analysis of trait relationships and genotype performance under contrasting water regimes. Principal Component Analysis (PCA) biplots showing the distribution of 18 rice genotypes (15 advanced lines and 3 checks) and their associated traits under (**a**) well-watered (WW) and (**b**) water-deficit (WD) conditions. Genotypes are represented by colored dots, while traits are shown as vectors. Pearson correlation matrices displaying the relationships among physiological and agronomic traits under (**c**) WW and (**d**) WD conditions are presented below the corresponding PCA plots. Correlation coefficients are color-coded from blue (negative) to red (positive), with *, **, and *** indicating significance at *p* < 0.05, *p* < 0.01, and *p* < 0.001, respectively. GY: grain yield, TGW: 1000-grain weight, PL: panicle length, NPP: number of panicles per plant, PH: plant height, DTH: days to 50% heading, SC: stomatal conductance, LR: leaf rolling, RWC: relative water content, PC: proline content, POD: peroxidase, CAT: catalase activity: CAR: carotenoid content, Chla: chlorophyll a, Chlb: chlorophyll b

The combined application of PCA and correlation analysis elucidated distinct trait relationships and genotype responses in the physiological and yield performance of 15 rice advanced lines and 3 check genotypes under differing water regimes (Fig. 2). Under WW conditions, the first two principal components (PC1 and PC2) accounted for 45.0% of the total variation (PC1: 24.9%, PC2: 20.1%) (Fig. 2a). The PCA biplot revealed a broad distribution of traits, suggesting diverse responses among genotypes. Positive associations were observed among carotenoid content, chlorophyll b, and chlorophyll a, as well as between SC and TGW with grain yield (GY). Sterility percentage, PC, and PH were positively associated, while LR showed negative correlations with these characteristics. The correlation analysis confirmed these relationships, with GY exhibiting strong positive correlations with chlorophyll a (*r = *0.57*), NPP (*r = *0.53*), TGW (*r = *0.49*), and RWC (*r = *0.48*), and a negative correlation with sterility (*r = *−0.49*) (Fig. 2c). Additionally, TGW correlated positively with DTH (*r = *0.50*), while DTH showed a strong negative correlation with RWC (*r = *−0.73***). Chlorophyll a demonstrated a strong positive correlation with chlorophyll b (*r = *0.75***). Genotypes including Giza-177, Sakha-107, L1, L5, and L15 clustered in the positive PC1 region, indicating superior performance for yield-related traits. IRAT-170 and L8 appeared as outliers, suggesting unique trait combinations.

Under WD conditions, the trait relationships were modified, with PC1 and PC2 accounting for 30.7% and 16.3% of the overall variance, respectively (Fig. 2b). The increased variance explained by PC1 indicated a heightened impact of water stress on trait interrelationships. The most notable change was the strong positive correlation between sterility percentage and LR, which were inversely related to yield components, highlighting their potential as key indicators of drought stress susceptibility. Correlation analysis under WD conditions revealed that GY maintained strong positive correlations with chlorophyll a (*r = *0.48*), PC (*r = *0.52*), RWC (*r = *0.56*), NPP (*r = *0.51*), and TGW (*r = *0.54*), while negatively correlating with sterility (*r = *−0.53*) (Fig. 2d). TGW showed negative correlations with LR (*r = *−0.67**) and sterility (*r = *−0.56*), and sterility negatively correlated with RWC (*r = *−0.50*). Peroxidase activity (POD) positively correlated with carotenoid content (CAR) (*r = *0.52*), while CAR was positively correlated with both chlorophyll a (*r = *0.62**) and chlorophyll b (*r = *0.75***). Under water stress, carotenoid content, PH, and peroxidase activity formed a distinct cluster, while RWC, chlorophyll a, GY, PC, and 1000-grain weight exhibited significant close interrelations, underscoring their cumulative significance in sustaining yield under stress. In addition, DTH and NPP showed inverse correlations with stress response and yield traits. The genotypes IRAT-170 and Sakha-107 demonstrated superior performance in water-limited environments, as evidenced by their positioning within the biplot. Conversely, Giza-177 exhibited substantial positional alteration relative to well-irrigated conditions, indicating increased susceptibility to water stress.

### Path analysis of rice traits on grain yield

**Fig. 3:**
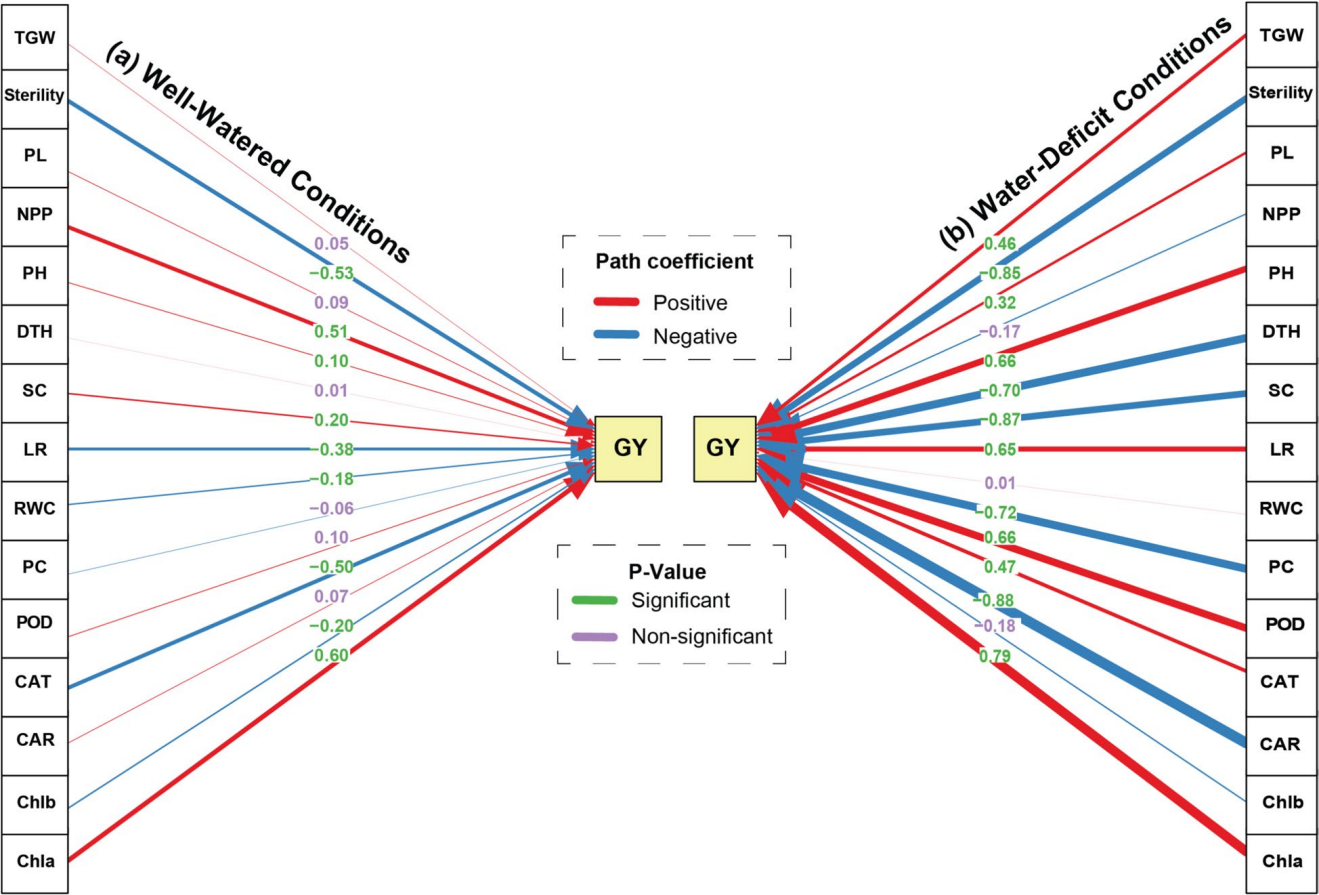
Path analysis diagram illustrating the relationships between various traits and grain yield (GY) in rice under WW (**a**) and WD (**b**) conditions. TGW: 1000-grain weight, PL: panicle length, NPP: number of productive tillers, PH: plant height, DTH: days to heading, SC: stomatal conductance, LR: leaf rolling, RWC: relative water content, PC: protein content, POD: peroxidase, CAT: catalase, CAR: carotenoid, Chla: chlorophyll a, Chlb: chlorophyll b. Path coefficients are color-coded: positive coefficients are indicated in blue and negative coefficients in red. Significant paths (*p* < 0.05) are shown in green and non-significant paths (*p* > 0.05) in purple. The width of the arrows represents the magnitude of the path coefficients, providing a visual representation of the strength of each relationship

Our path analysis revealed distinct patterns of trait influences on GY under WW and WD conditions in rice (Fig. 3). Under WW conditions (Fig. 3a), Chla exhibited the strongest positive direct effect on GY (β = 0.602, *p* < 0.001), followed by NPP (β = 0.513, *p* < 0.001). Conversely, sterility showed the most substantial negative impact (β = −0.526, *p* < 0.001), closely followed by CAT (β = −0.504, *p* < 0.001). Other significant positive influences included SC (β = 0.197, *p* < 0.001) and PH (β = 0.102, *p* < 0.001), while LR (β = −0.385, *p* < 0.001) and Chlb (β = −0.198, *p* < 0.001) demonstrated negative effects. Interestingly, RWC showed a small but significant negative association with GY (β = −0.183, *p* < 0.01).

The path analysis coefficients have dramatically changed under WD conditions (Fig. 3b). Chla maintained its positive influence but with a markedly stronger effect (β = 0.79, *p* < 0.001). Carotenoid content, which was non-significant under WW conditions, emerged as a strong negative predictor of GY (β = −0.88, *p* < 0.001). PC and DTH also showed strong negative associations (β = −0.72 and β = −0.70 respectively, both *p* < 0.001) under water stress. Peroxidase activity and PH demonstrated significant positive effects (β = 0.66 and β = 0.66 respectively, both *p* < 0.001). Notably, catalase activity shifted from a negative to a positive influence (β = 0.470, *p* < 0.001), while spikelet count reversed from positive to negative (β = −0.867, *p* < 0.001). Some traits, such as NPP and Chlb, lost their significant associations with GY under WD conditions, while others like PL and TGW gained significance. The relationship between RWC and GY became non-significant under water stress.

## Discussion

These findings of ANOVA among genotypes, environments, and their interactions for most traits highlight the complex nature of drought response mechanisms and ensure that breeding programs for improving drought tolerance should take both genetic as well as environmental factors into account. There was a significant effect of environment and genotype for both chlorophylls in leaves, which corroborates results from previous works showing changes in leaf pigments under drought conditions [41]. This pronounced genotype × environment interaction for chlorophyll content implies that plant genotypes coping with drought may differ in the strategy to keep their photosynthesis intact. Also, the significant differences in carotenoid content among the environments and genotypes were evident, suggesting that these pigments could play a supplementary role as plant protection compounds under drought stress [42]. Carotenoids are vital for photoprotection but also act as antioxidants during stress responses [43].

The differences in the activities of antioxidant enzymes (catalase and peroxidase) with significance among treatments and genotypes as well as their interactions have profound implications for response to drought stress by the oxidative defense system. This was in agreement with earlier studies that reported an enhancement of antioxidant enzyme activities as a defense mechanism to scavenge the reactive oxygen species produced under drought stress [44]. Also, the differential responses among genotypes, particularly the high activities observed in IRAT-170 and L5 under WD conditions, suggest genotype-specific enhanced antioxidant defense mechanisms.

The findings of the current study indicated an increase in proline content under water-deficit conditions across all genotypes, with concentrations varying from 0.61 to 0.87 mg g⁻^1^ FW under WD, in contrast to 0.37 to 0.74 mg g⁻^1^ FW under WW conditions. The increase in proline accumulation due to water stress validates its function as a compatible osmolyte in osmotic adjustment mechanisms among various rice genotypes. The interpretation of proline accumulation as an indicator of stress tolerance necessitates careful consideration in conjunction with actual performance metrics, rather than being assessed as an isolated parameter. The findings elucidate the complexity of proline's involvement in stress responses, reinforcing the expanding literature that challenges its conventional designation as a direct marker of tolerance. Analysis of proline accumulation patterns in relation to stress tolerance, as indicated by grain yield performance, revealed distinct responses. Stress-tolerant genotypes (L5 and L11) exhibited moderate increases in proline levels under water-deficit conditions, while sustaining superior grain yield performance, indicating effective osmotic adjustment rather than stress-induced distress [45]. In contrast, stress-sensitive genotypes like Giza-177 demonstrated increased proline accumulation despite suboptimal grain yield performance, likely indicating enhanced stress perception and cellular damage rather than effective adaptive tolerance mechanisms. The L8 genotype exhibited the highest proline concentration, measuring 0.74 mg g⁻^1^ FW, even in well-watered conditions, while also demonstrating robust physiological performance under stress. This indicates that constitutive proline synthesis may play a protective role. The observed patterns indicate that moderate increases in proline levels among high-performing genotypes may serve as an effective mechanism for osmotic adjustment, whereas excessive proline accumulation in low-performing genotypes is likely indicative of heightened stress sensitivity.

The RWC, LR, and SC traits are typically related to drought avoidance mechanisms in rice [46]. RWC decreased under water-stressed conditions for all genotypes, as expected, however, Sakha-107 maintained the highest RWC under both water scenarios, suggesting superior water retention capacity. The lower LR scores observed in L8 under WD conditions, combined with its maintenance of high chlorophyll content, suggest that this genotype may employ an effective drought tolerance strategy. IRAT-170 preserved the highest level under such stress conditions, implying that this variety could achieve drought tolerance by maintaining gas exchange rates by preserving higher transpiration levels [47]. DTH showed significant variations for environments and genotypes, but not for their interaction. The DTH was generally reduced under WD conditions, which is consistent with drought escape mechanisms often observed in cereals (Farooq et al., 2009). Such a response could be advantageous in rice breeding programs aiming to develop varieties with predictable flowering and maturity periods (Iqbal et al., 2019). The consistent early flowering of Sakha-107 under both conditions suggests a genetically determined short life cycle, allowing the plant to complete its life cycle before severe water stress occurs. PL and the NPP decreased due to water stress, as indicated by the results; the two traits are known to be sensitive to water stress during the reproductive stage [48]. The IRAT-170 and L3 genotypes were able to keep rather high values of these characters under WD conditions indicating certain means under drought stress. Similar results have been noted by [49] They concluded that the number of panicles is one of rice's most critical yield components affected by drought stress. Also, the high sterility % under WD is a phenomenon well-documented in the behavior of rice and is manifested more through the pollen sterility coupled with reduced seed set [50], evidencing that the rice reproduction system is very sensitive to drought [51]. On the other hand, L8 although being lower in sterility under WD conditions and which has been previously described to maintain high chlorophyll content and low level of LR under water stress, may be considered a drought-tolerant genotype.

Importantly, yield traits, including TGW, differed significantly among environments, genotypes, and their interaction, indicating that grain filling is affected by drought stress and that genotypes respond differently to this stress. This trait is crucial for final yield determination and is sensitive to water deficit during the grain-filling period [52, 53]. The highest TGW for the Sakha-107 cultivar exhibited under the WD conditions also indicates that it could possess a strong and efficient grain-filling process under stressful conditions which may add to the stability of the yield. Notably, GY, the primary quantitative trait under the plant breeding perspective, exhibited significant variations across the years, environments, and genotypes, with a strong G × E effect. The year effect implies that environmental variations interfere with yield within the years, and hence there is a correlation with the productivity of rice grains, a fact supported by prior long-term investigations on rice yield stability [54]. Interestingly, genotype L11 retained the highest GY in water-stressed environments and can be recommended for elaborate investigation on the mechanisms that help plants withstand the detrimental effects of drought.

Understanding the genetic variability of physiological, agronomic, and yield traits under WW and WD conditions is crucial for developing drought-tolerant rice varieties. Key observations included increased genetic and phenotypic variance in traits such as chlorophyll a and b, peroxidase, and RWC under WD conditions, highlighting their potential for selection in breeding programs aiming at drought tolerance. These traits exhibited significantly higher GCV under WD conditions, suggesting they are critical for maintaining photosynthetic efficiency and mitigating oxidative stress during water scarcity [55]. Conversely, traits like catalase activity and LR showed decreased genetic variability under drought, indicating a more constrained genetic response and potentially limiting their utility in drought-resilience breeding. However, the increased variability in peroxidase activity underscores its role in oxidative stress management under drought conditions [56]. Each of NPP and PL demonstrated increased genetic variability under drought, suggesting their importance for yield stability in stressed environments. The significant rise in genetic variability for sterility percentage under drought stresses indicates the sensitivity of reproductive processes to water deficit, directly impacting GY [57]. While GY itself showed a slight decrease in genotypic variance under drought, the increase in both GCV and PCV indicates a broader phenotypic response range. This suggests the potential for selecting genotypes with stable performance across varying environmental conditions [58]. By understanding the genetic basis and variability of these traits under stress, breeders can more effectively develop resilient rice varieties capable of sustaining productivity under adverse conditions.

The present study exploited PCA to establish insights into the genotype performance of rice under contrasting water conditions for physiological and agronomic traits. PCA is an effective method for identifying genetic components that influence complex characteristics [59], as it elucidates the interrelationships of the intricate traits and the responses of genotypes to varying water conditions on a multivariate level. Thus, in the current study, PCA illustrated different patterns of trait associations and genotype responses under WW and water deficit conditions, indicating that the rice genotypes can show variation depending on water supply. The positive association between SC, 1000-grain weight, and GY agrees with earlier studies and highlights effective gas exchange as critical for yield under a favorable environment [60]. IRAT-170 and L8 were observed as outlier positions in the PCA biplot, revealing they possess unique trait combinations that may help to identify novel alleles for breeding programs that aim to increase yield potential under stress conditions.

The change in associations between traits and the higher proportion of trait variation captured in association with PC1s may imply a major reordering of plant physiological and agronomic responses in response to the perceived water stress conditions. The remarkable positive relationship between sterility (%) and LR, and negative relationships with yield components, underscore the importance of these selection traits for assessing drought stress, as previously observed [1], and the vital importance of maintaining reproductive potential. The alignment of carotenoid levels, PH, and peroxidase activity, in the same direction in PCA under WD environments indicates a coordinated response that combines antioxidant defense mechanisms and growth regulation. The strong association among water content chlorophyll level, GY, PC, and 1000-grain weight underscore their significance in sustaining yield during stressful conditions. Additionally, negative correlations of DTH and NPP with stress-response and yield-related traits imply that coming into flower earlier and producing fewer tillers could be a way some genotypes adapt to water deficit. This is in line with observations supporting drought escape and water conservation strategies documented in other cereals [61]. Interestingly, PCA findings indicated a contrasting view for the Giza-177 cultivar under both contrasting water scenarios, representing a significant shift and posing possibilities to their sensitivity to changing water supply.

The results of our path analysis elucidate the dynamic nature of trait influences on GY in rice under varying water conditions, suggesting the critical role of both physiological and morphological traits in depicting such crop productivity. Under WW conditions, our findings corroborate the significance of chlorophyll a as a primary positive determinant of GY. This strong positive effect aligns with the role of Chla in photosynthetic efficiency, which is pivotal for biomass accumulation and grain filling. Conversely, sterility exhibited the most pronounced negative impact on GY, which indicates the critical importance of successful fertilization and seed set for yield realization. Catalase activity, typically associated with oxidative stress mitigation, surprisingly showed a significant negative effect, suggesting a potential trade-off between stress response and yield under non-stress conditions [62]. Both SC and PH positively influence GY, contributing to efficient water use and gas exchange.

Under WD conditions, the influence of Chla was amplified, highlighting its critical role in maintaining photosynthetic efficiency under stress. This finding is consistent with research indicating that maintaining high Chla levels can be a key adaptive trait for drought tolerance [63]. The emergence of carotenoid content as a strong negative predictor suggests that excessive accumulation of carotenoids might indicate stress severity beyond a beneficial threshold, aligning with studies that show a complex relationship between carotenoid levels and stress adaptation [64]. Interestingly, traits such as PC and DTH showed strong negative associations with GY under WD conditions may reflect the trade-offs between stress resistance mechanisms and reproductive success. Similarly, the shift of catalase activity from a negative to a positive influence under stress conditions underscores its importance in mitigating oxidative damage when plants are subjected to drought. The shift from a positive to a negative effect between SC and GY, depending on water availability, highlights its complex role. Elevated SC benefits yield under ample water but can cause excessive water loss and reduced yield under WD conditions, showing its potential harm under water-limited conditions. The differential trait influences under WW versus WD conditions highlight the need for targeted approaches to optimize yield based on specific environmental contexts. Our findings suggest that breeding programs should prioritize traits such as Chla, NPP, and SC for WW environments while focusing on maintaining Chla, optimizing carotenoid content, and enhancing peroxidase activity under drought conditions to improve resilience and yield stability.

## Conclusion

The present study provides comprehensive insights into the complex mechanisms of drought tolerance in rice through a detailed analysis of physiological and agronomic traits under two contrasting water regimes. Significant genotype × environment interactions were observed for most traits, highlighting the plasticity of rice genotypes in response to water availability and emphasizing the vital role of multi-environment testing in drought tolerance breeding programs. Our findings reveal differential responses in photosynthetic pigments, antioxidant enzyme activities, and osmolyte accumulation among genotypes under drought stress, indicating diverse strategies for maintaining cellular function and mitigating oxidative damage. Notably, genotypes such as IRAT-170, Sakha-107, and L5 demonstrated superior drought tolerance traits and yield stability under WD conditions, making them valuable genetic resources for future breeding efforts. Multivariate analyses uncovered complex trait associations, underscoring the interconnected nature of physiological and agronomic responses to drought stress. The clustering patterns observed in PCA highlight the importance of considering multiple traits simultaneously in breeding for drought tolerance. Also, the identification of promising advanced lines (e.g., L5, L10) with enhanced drought tolerance traits warrants further genetic investigation to elucidate the molecular basis of their stress resilience. These genotypes may offer novel alleles for improving both yield potential and stress tolerance in rice breeding programs.

Some future strategies should focus on incorporating drought tolerance mechanisms from resilient genotypes like IRAT-170 and Sakha-107 into high-yielding backgrounds and utilizing genomic tools to unravel the genetic basis of observed trait associations and facilitate marker-assisted selection for drought tolerance. Integrating these future perspectives with the outputs of the current study enables the rice breeders to accelerate the development of rice varieties that combine high yield potential with enhanced resilience to water-limited conditions. Such efforts are crucial for addressing the challenges posed by climate change and water scarcity in global rice production systems.

## Supplementary Information

Supplementary Material 1.

Supplementary Material 2.

Supplementary Material 3.

Supplementary Material 4.

## Data Availability

All the data are available in the manuscript and with Correspondence authors.

## Abbreviations

Chla: Chlorophyll a
Chlb: Chlorophyll b
CAR: Carotenoid
CAT: Catalase
POD: Peroxidase
PC: Proline content
RWC: Relative water content
LR: Leaf rolling
SC: Stomatal conductance
DTH: Days to 50% heading
PH: Plant height
NPP: Number of panicles per plant
PL: Panicle length
TGW: 1000-Grain weight
GY: Grain yield
WW: Well-watered
WD: Water-deficit
