# Supplementary material for: Genetic variability in physiological and agronomic traits of newly developed rice lines under well-watered and water-deficit conditions
Source: BMC Plant Biol. 2025 Oct 2;25:1291. doi: 10.1186/s12870-025-07436-3 (PMC12490075; doi:10.1186/s12870-025-07436-3)
Supplement: Supplementary file 1 — Supplementary Material 1. [file 12870_2025_7436_MOESM1_ESM.docx]

**Table S1. Some physical and chemical soil characteristics of the experimental sites during the 2022 and 2023 growing seasons.**

| Soil characteristics | 2022 | 2023 |
| --- | --- | --- |
| Soil texture (%) | Clay | Clay |
| Sand (%) | 21.43 | 21.2 |
| clay (%) | 49.15 | 48.5 |
| Silt (%) | 29.42 | 30.3 |
| EC (dSm^-1^) | 2.22 | 2.26 |
| pH (1: 2.5 water suspension) | 8.13 | 8.11 |
| Organic matter | 1.36 | 1.38 |
| Available P (ppm) | 12.16 | 12.36 |
| Exchangeable K (ppm) | 443.8 | 442.8 |
| Total N (ppm) | 582 | 583 |
| Available K Mg Kg^-1^ | 342 | 349 |
| Cations (meq/L.) |  |  |
| Mg^++^ | 2.33 | 2.43 |
| Ca^++^ | 5.36 | 5.22 |
| K^+^ | 1.4 | 1.25 |
| Na^+^ | 12.2 | 13 |
| Anions (meq/L) |  |  |
| Cl^-^ | 15.2 | 15.76 |
| HCO3- | 3.71 | 4.22 |
| CO3^—^ | 0 | 0 |
| SO4^- -^ | 2.38 | 1.92 |
