# Supplementary material for: Genetic variability in physiological and agronomic traits of newly developed rice lines under well-watered and water-deficit conditions
Source: BMC Plant Biol. 2025 Oct 2;25:1291. doi: 10.1186/s12870-025-07436-3 (PMC12490075; doi:10.1186/s12870-025-07436-3)
Supplement: Supplementary file 2 — Supplementary Material 2. [file 12870_2025_7436_MOESM2_ESM.docx]

| **Genotype** | **Grain yield** | **Rank** | **1000-grain weight** | **Rank** | **Days to 50% heading** | **Rank** | **Plant height** | **Rank** | **No. of panicles** | **Rank** | **Panicle length** | **Rank** | **Sterility** | **Rank** |
| --- | --- | --- | --- | --- | --- | --- | --- | --- | --- | --- | --- | --- | --- | --- |
| **L11** | 9.88 | **1** | 24.72 | **3** | 99.33 | 5 | 76.35 | 9 | 15.3 | 14 | 23.24 | 2 | 4.64 | 17 |
| **L5** | 9.63 | **2** | 22.73 | **12** | 97.9 | 7 | 84.62 | 5 | 18.61 | 5 | 20.62 | 6 | 9.66 | 13 |
| **Sakha-107** | 9.21 | **3** | 25.37 | **1** | 88.13 | 17 | 89.06 | 3 | 18.69 | 4 | 20.11 | 7 | 14.88 | 4 |
| **L6** | 8.88 | **4** | 23.03 | **10** | 95.87 | 10 | 75.85 | 10 | 16.06 | 11 | 18.64 | 12 | 9.94 | 10 |
| **L3** | 8.59 | **5** | 23.13 | **8** | 93.62 | 14 | 78.78 | 7 | 22.48 | 1 | 16.8 | 17 | 5.76 | 16 |
| **L8** | 8.19 | **6** | 25.27 | **2** | 101.98 | 2 | 74.03 | 13 | 15.63 | 13 | 21.5 | 5 | 4.04 | 18 |
| **L15** | 8.14 | **7** | 23.15 | **7** | 97.7 | 8 | 106.38 | 2 | 16.98 | 9 | 21.75 | 3 | 12.76 | 6 |
| **L4** | 8.05 | **8** | 23.45 | **6** | 99.68 | 4 | 71.8 | 16 | 17.96 | 7 | 18.84 | 11 | 9.7 | 12 |
| **L9** | 8.03 | **9** | 23.05 | **9** | 94.93 | 11 | 87.96 | 4 | 17.51 | 8 | 18.34 | 13 | 9.92 | 11 |
| **L2** | 7.9 | **10** | 24.2 | **4** | 93.67 | 13 | 69.78 | 17 | 19.32 | 2 | 21.73 | 4 | 12.17 | 7 |
| **L10** | 7.89 | **11** | 19.8 | **17** | 98.5 | 6 | 81.73 | 6 | 18.48 | 6 | 19.87 | 9 | 11.25 | 8 |
| **L1** | 7.68 | **12** | 24.05 | **5** | 92.81 | 16 | 68.79 | 18 | 16.31 | 10 | 17.1 | 16 | 10.29 | 9 |
| **L7** | 7.34 | **13** | 21.02 | **16** | 96.94 | 9 | 73.33 | 14 | 18.81 | 3 | 15.11 | 18 | 8.98 | 15 |
| **IRAT-170** | 7.28 | **14** | 22.78 | **11** | 99.99 | 3 | 106.5 | 1 | 15.64 | 12 | 23.62 | 1 | 15.23 | 3 |
| **L12** | 7.2 | **15** | 21.9 | **14** | 93.6 | 15 | 76.61 | 8 | 14.94 | 15 | 20.09 | 8 | 9.5 | 14 |
| **Giza-177** | 6.63 | **16** | 17.37 | **18** | 87.11 | 18 | 74.7 | 12 | 12.33 | 18 | 17.21 | 15 | 21.18 | 1 |
| **L13** | 6.13 | **17** | 21.82 | **15** | 94.75 | 12 | 75.22 | 11 | 13.95 | 17 | 19.72 | 10 | 12.82 | 5 |
| **L14** | 4.65 | **18** | 21.92 | **13** | 106.34 | 1 | 72.25 | 15 | 14.15 | 16 | 17.5 | 14 | 15.36 | 2 |

**Table S2. Ranking of 18 rice genotypes based on agronomic traits performance under water-deficit conditions**
