# Supplementary figures and images for: Genetic variability in physiological and agronomic traits of newly developed rice lines under well-watered and water-deficit conditions
Source: BMC Plant Biol. 2025 Oct 2;25:1291. doi: 10.1186/s12870-025-07436-3 (PMC12490075; doi:10.1186/s12870-025-07436-3)

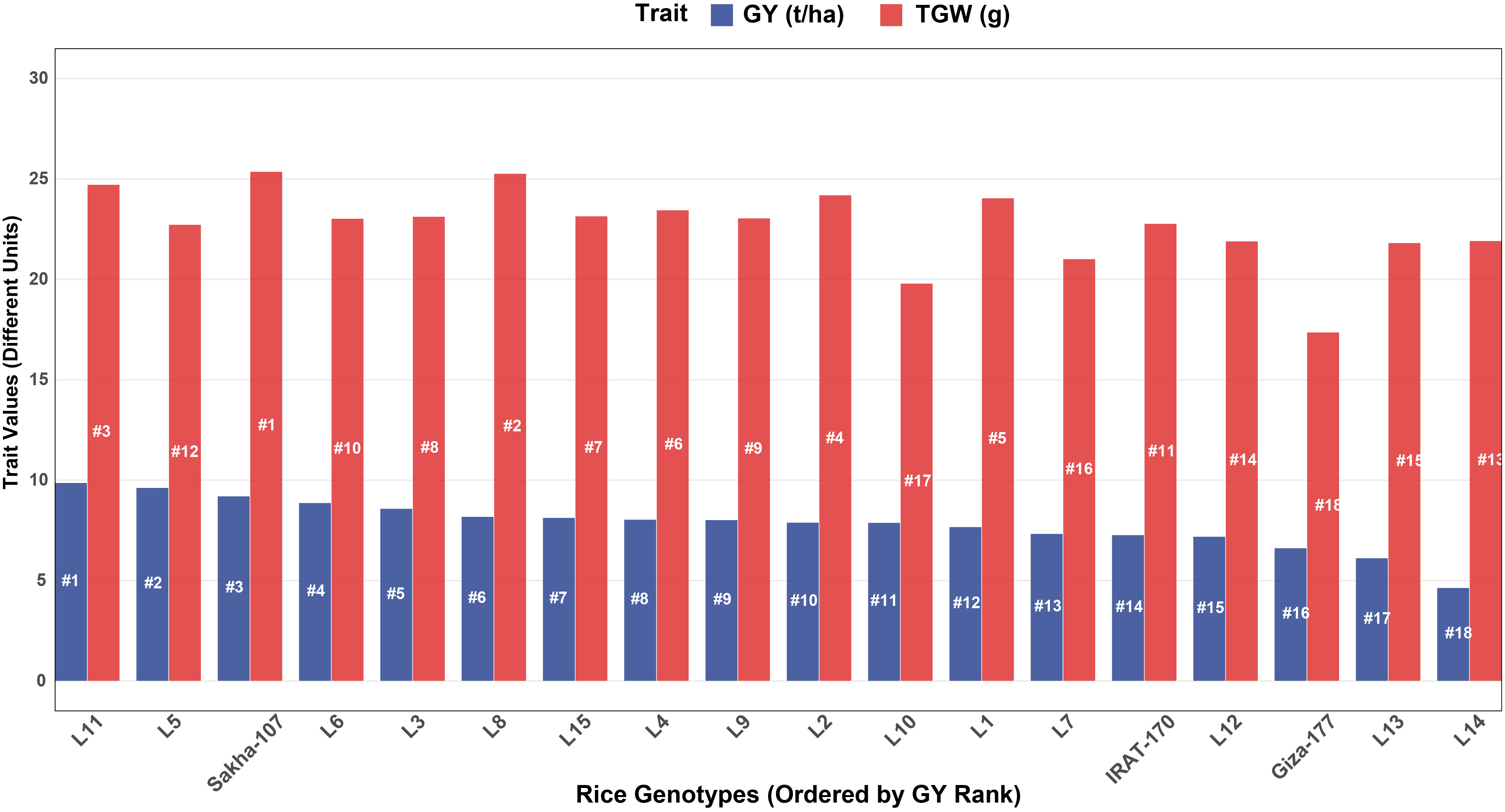

Supplement: Supplementary file 3 — Supplementary Material 3. [file 12870_2025_7436_MOESM3_ESM.png]
