# Supplementary material for: Genetic variability in physiological and agronomic traits of newly developed rice lines under well-watered and water-deficit conditions
Source: BMC Plant Biol. 2025 Oct 2;25:1291. doi: 10.1186/s12870-025-07436-3 (PMC12490075; doi:10.1186/s12870-025-07436-3)
Supplement: Supplementary file 4 — Supplementary Material 4. [file 12870_2025_7436_MOESM4_ESM.pdf]

Field Experiment Layout

Randomized Complete Block Design (RCBD) with Three Replications

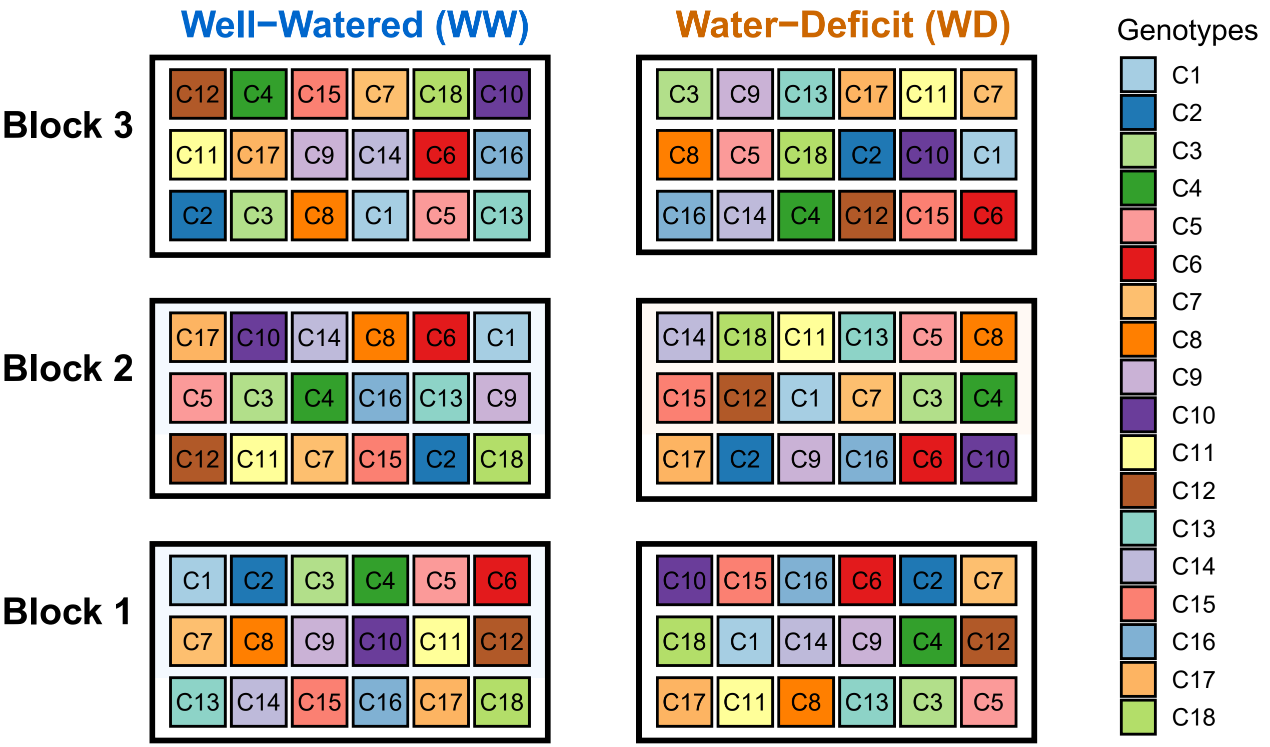

Plot size: 1.2 m × 5 m (6 m^2), containing 6 rows with 20 cm spacing
Each row is 5 m long with plants spaced 20 cm apart (approx. 25 plants per row)
